# Surgical risk assessment for gynecological oncologic patients

**DOI:** 10.4274/tjod.galenos.2019.93584

**Published:** 2019-10-10

**Authors:** Çağlayan Biçer, Jalal Raoufi, Serhan Can İşcan, Mehmet Güney, Evrim Erdemoğlu

**Affiliations:** 1 Süleyman Demirel University Faculty of Medicine, Department of Obstetrics and Gynecology, Isparta, Turkey; 2 Süleyman Demirel University Faculty of Medicine, Department of Obstetrics and Gynecology, Division of Gynecologic Oncology, Isparta, Turkey

**Keywords:** Gynecological oncology, metoprolol, upper abdominal surgery, surgical risk assessment

## Abstract

**Objective:**

Preoperative surgical risk assessment is important in terms of postoperative morbidity and mortality. Therefore, it is necessary to evaluate the efficacy and safety of these surgeries via an ideal risk assessment model, and reduce risks via applying some findings (for instance, perioperative beta-blockers). There are some risk assessment systems, but these have generally not been verified for patients with gynecologic cancer. The aim of this study was to assess the risk of surgery for gynecological oncologic patients and suggest an easy risk assessment model and risk reduction by applying our findings.

**Materials and Methods:**

We retrospectively analyzed 258 gynecologic patients with cancer. Age, diagnosis, staging, performance scale, metoprolol use, heart, renal diabetes, Chronic Obstructive Pulmonary disease, diabetes, operation type and length, carcinoma antigen 125, ascites, albumin, surgical procedure, hospitalization length, and complications were recorded.

**Results:**

Of the 258 patients, 173 patients (67.1%) had no complications, 43 patients (16.7%) had one and 42 patients (16.3%) had two or more complications. The most common complication was the acid-base imbalance (14%), followed by urinary tract infection (9.7%). Parameters associated with complications were performance status, ascites, operating length, metoprolol use, and upper abdominal surgery. In our proposed scoring model with a total score range 0-23, cut-off value points for both the presence and rate of complications was found as >5.

**Conclusion:**

In gynecological patients with cancer, the addition of metoprolol use and upper abdominal surgery within preoperative risk assessment evaluation parameters are significantly effective in predicting the rate and severity of complications. Moreover, we have suggested a simple, practical, and convenient scoring model for this evaluation.

**PRECIS:** For preoperative risk assessment in gynecologic cancers, a simple and practical scoring model is recommendable. Moreover, the addition of metoprolol use and upper abdominal surgery improve the accuracy of these programs.

## Introduction

In 2016, approximately 105.000 new cases of gynecologic malignancies were estimated in the United States of America^(1)^. Two-thirds of these cases will undergo surgery^(2)^. Usually, complete tumor resection is a goal, and these surgeries may be expanded as upper abdominal surgery such as diaphragmatic peritoneal resection, splenectomy, and segmental liver resection according to the patients’ condition and diagnosis^(3)^. It is necessary to evaluate the efficacy and safety of these surgeries because of the association between extensive surgical procedures and postoperative morbidity and mortality^(4)^. Furthermore, the initiation of postoperative chemotherapy may lag due to these complications^(4)^. For example, extensive debulking ovarian cancer surgery to no gross residual tumor may be accompanied by major complications in about 50% of these patients, especially in older patients, the risks of mortality and morbidity are greater^(5)^. There are some risk assessment systems for surgical risk assessment; however, generally, the predictive value of these systems has not been verified for patients with gynecologic cancer^(6,7)^. Therefore, a risk scoring model study was performed to predict major complications in patients with ovarian cancer who underwent laparoscopic interventions before primary debulking surgery. In the validation population, observed risk and predicted risks were 16.7% and 17.8%, respectively. The major contribution of this study was to provide a preoperative tool to predict outcomes^(5)^. An ideal risk assessment model would be simple, reproducible, authentic and correct, objective, and accessible to all patients, and especially able to perform personalized assessments of patients according to the use of patient-specific characteristics^(5,8)^. Furthermore, ideally, it should be low-cost and feasible to perform at the bedside^(8)^. Thus, as physicians, our endeavor is to perform a simple and practical risk assessment to prevent complications and assure decreased peri-operative healthcare costs and postoperative morbidity and mortality. Notably, when compared with other elections, conservative treatment or neoadjuvant chemotherapy can be performed instead of upfront surgical treatment^(5,9)^. Several studies have shown that the use of perioperative beta-blockers (metoprolol was shown as more suitable), reduces mortality in both cardiologically high and low-risk operations^(10,11,12,13)^. The aim of this study was to assess the risk of surgery for gynecological oncologic patients and to suggest an easy risk assessment model that was feasible to perform at the bedside, and reduce risk of postoperative complications by applying our data and findings.

## Materials and Methods

### Study design

We retrospectively analyzed 258 patients with gynecologic cancer who underwent surgery between 2008 and 2017, and whose complete data were available. In our center, Eastern Cooperative Oncology Group (ECOG) performance status (PS) score findings and presence of systemic diseases are routinely determined and noted in patient files during the hospitalization of the patients. Additionally, we measure electrolytes and draw blood gases for all patients before and after surgery; we analyzed these data.

### Study variables

The evaluated parameters were stage of primary disease according to the International Federation of Gynecology and Obstetrics (FIGO) (stage 1-2: non-disseminated, stage 3-4: disseminated), age (<65 or ≥65 years),^(14)^ PS scale (ECOG),^(15) ^carcinoma antigen 125 (CA-125) (<500, 500-1000, >1000 IU/dL), amount of ascites (<500, 500-1000, >1000 mL), diabetes (no, <10 years, >10 years,) according to a few studies about duration-related diabetes morbidity,^(16,17) ^Chronic Obstructive Pulmonary disease (COPD), heart disease (arrhythmia, heart failure), renal disease (renal failure, others), preoperative albumin (<3 or >3 g/dL),^(7)^ surgical procedures including major pelvic surgery,^(18)^ and upper abdominal surgery,^(3)^ the total surgical time^(2)^ (<4 or ≥4 hours), metoprolol use,^(10,13)^ operation intent (primary, recurrent), and the length of hospital stay (Table 1). We categorized these parameters as the above-mentioned references^(2,3,10,11,12,13,14,15,16,17,18)^. Complications were electrolyte imbalance (hypernatremia, hyponatremia, hypokalemia hyperkalemia, hypocalcemia, hypercalcemia, hypermagnesemia, hypomagnesemia), acid-base imbalance, pneumonia, venous thromboembolism, death, surgical site infection, renal failure, postoperative transfusion, and urinary tract infection (UTI) (Table 2). Diagnosis was made histopathologically. The patient who underwent the first surgery was recorded as primary and the others were as recurrent. We recorded scoring system parameters using specific criteria. The stage was determined according to the FIGO criteria. PS was recorded according to the ECOG score, which is classified from 0 to 4. Patents who are ECOG 0 have no limitations, ECOG 1 has mild limitation in exhausting activity, ECOG 2 is partially dependent, and ECOG 3 is capable of limited self-care. Patients wo are ECOG 4 cannot resume self-care without continuous support^(15)^. Upper abdominal surgery includes diaphragmatic peritoneal resection, splenectomy, pancreatectomy, gastrectomy, segmental liver resection, and biliary surgery. Major pelvic surgery encompasses radical hysterectomy, pelvic lymph node dissection, pelvic exenteration, and debulking surgery^(3,18)^. In our center, within a certain time period, based on previous studies,^(10,11,12,13)^ 8.5% of patients received metoprolol two days prior to surgery and continued one week after surgery. Postoperative complications including acid-base imbalance, electrolyte imbalance, pneumonia, surgical site infection, and renal failure were defined and recorded according to the Common Terminology Criteria for Adverse Events^(19)^. Additionally, we recorded the presence of complications, number of complications, and total score. The study was approved by the Süleyman Demirel University Local Ethics Committee (approval number: 164, date: 28.09.2016). Additionally, consent forms were routinely completed by patients at the time of hospitalization.

### Statistical Analysis

Statistical analyses were performed using the Medcalc Software (version 16.8). Forward regression analysis was used to identify the predictive scoring parameters. P values of 0.05 or less were regarded as statistically significant. We used multiple regression analysis to predict the number of complications, presence of complications, and length of hospital stay. We assessed the area under the curves (AUC) of the receiver operating characteristic (ROC) curve for predicting models of risk scoring. To assess the optimal cut-off point, Youden’s index (sensitivity + specificity-1) was used.

## Results

**Figure 1:**
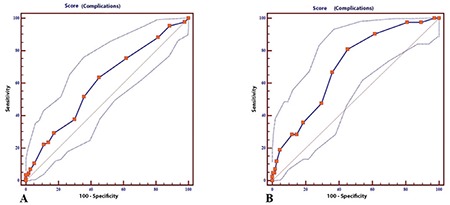
Performance assessment of the scoring system to predict the complications using the receiver operating characteristic curve. A) For the presence of complications, the area under curve (AUC) was found as 0.60 (thick and quadratic curve) with 95% confidence interval (CI) 0.54-0.66 (dotted curves for lower and upper bound of 95% CI). Youden’s index was 0.18 (cut-off value for the count of points in the model >5), p=0.005, B) For the count of complication, the AUC was found as 0.70 (thick and quadratic curve) with 95% CI: 0.64-0.75 (dotted curves for lower and upper bound of 95% CI). Youden’s index was 0.35 (cut-off value for the count of points in the model >5), p<0.0001

The mean age of the entire population was 58.8±10.9 years, where 77 patients (29.8%) were aged ≥65 years. The median total surgical time was 4 (range, 0.5-13) hours. The number of patients who underwent surgery for primary disease was 214 (82.9%) and for the recurrent disease it was 44 (17.1%). The majority of the patients were early stage (61.2%). In our study, 157 patients (60.9%), 85 patients (32.9%) and 16 patients (6.2%) underwent surgery for uterine cancer, ovarian cancer, and cervical cancer, respectively. We follow up the patients according to enhanced recovery after surgery protocols^(20,21) ^and the median length of hospital stay was 9 (range, 1-65) days. Our center is a reference center and accepts complicated patients; for instance, 201 patients (77.9%) underwent major pelvic surgery, so the median length of hospital stay was found as 9 days. When PS was evaluated, there were 183 patients (70.9%) with ECOG PS <2, 43 patients (16.7%) with an ECOG PS of 2, and 32 patients (12.4%) with ECOG PS ≥3 (the majority of them were under ECOG 2). In the analysis of complications, the majority of patients (173 patients, 67.1%) had no complications, 43 patients (16.7%) had one complication, and 42 patients (16.3%) had ≥2 complications. The most common complication was acid-base imbalance (14%), followed by UTI (9.7%) (Table 2). In multiple regression analysis, ECOG (p=0.02), ascites (p<0.01), total surgical time (p<0.0001), metoprolol use (p<0.0001), and upper abdominal surgery (p<0.0001) were found to be significantly effective for predicting complications (Table 3). ECOG score (p<0.001), presence of ascites (p<0.01), diabetes (p<0.01), major pelvic surgery (p<0.04), total surgical time (p<0.0004), metoprolol use (p<0.001), and upper abdominal surgery (p<0.001) were also found to be significantly correlated with the number of complications (Table 4). We assessed the performance of the scoring system using the ROC curve for estimating the presence of complications (Figure 1A). Finally, we evaluated the estimated count of complications (>1) using the ROC curve according to the scoring system (Figure 1B). In our scoring model, the total score range was between 0-23. For the presence of complications, the AUC was found as 0.60 with 95% confidence interval (CI) 0.54-0.66 and the Youden’s index was 0.18; the cut-off value in the model was >5, p=0.005. For the complications count, the AUC was found as 0.70 with 95% CI 0.64-0.75, and the Youden’s index was 0.35; the cut-off value in the model was >5, p<0.001.

## Discussion

### Main findings

Our study demonstrates that preoperative metoprolol use decreases and upper abdominal surgery increases the risk and number of postoperative complications in gynecological cancers. Additionally, other parameters that showed an association with postoperative complications and significance in our scoring system were the stage of the disease, ECOG, ascites, major pelvic surgery, total surgical time, and diabetes.

### Results of the study in the context of other observations

Similar to previous studies, the most common type of gynecologic cancer was uterine cancer, followed by ovarian and cervical cancer in this study^(1)^. In gynecologic cancers, prediction of postoperative complications is important because the incidence of these diseases is progressively increasing^(2,4)^. As a consequence, postoperative morbidity, mortality, and healthcare costs can be reduced through the prevention of postoperative complications. Previous studies have depicted that several parameters such as age, advanced stage, poor performance, ascites ≥1000, hypoalbuminemia, extended surgical time, and extensive surgery were associated with a higher risk of postoperative complications^(4,5,6,22,23,24,25,26,27)^. There are several studies about surgical risk assessment. Although some studies have been evaluated for gynecological cancers,^(2)^ generally they are non-specific in terms of gynecologic cancers or validated only for ovarian cancer^(25,26,27,28,29)^. On the other hand, several studies have shown that peri-operative beta-blockers use (metoprolol being more suitable and beneficial) was associated with reduced mortality among patients with high and low cardiac risk^(10,11,12,13)^. In our study, we evaluated the effect of metoprolol use on postoperative mortality and morbidity and it was significantly correlated with the prediction of complications (p<0.0001). Some studies investigated the role of extended surgery on postoperative mortality and morbidity. Patankar et al.^(26)^ reported that extended cytoreductive procedures were the strongest risk factor for complications in ovarian cancers. Conversely, Phillips et al.^(27)^ found that the number of surgical procedures was significantly correlated with an increased risk of major morbidity, and was a better predictor of major postoperative morbidity than the high-risk performance alone. Also, in the prediction of major complications, they found that ultra-radical surgery was less useful than any solitary gastrointestinal resection. They identified standard surgery as “total abdominal hysterectomy, bilateral salpingo-oophorectomy, omentectomy, pelvic and/or para-aortic lymphadenectomy, and bowel surgery outside the definition of ‘ultra-radical’ (localized colonic resection, non-multiple bowel resection)” and ultra-radical surgery as “diaphragmatic stripping, extensive peritoneal stripping, multiple resections of the bowel (excluding localized colonic resection), liver resection, partial gastrectomy, cholecystectomy, splenectomy”^(27)^. In this study, upper abdominal surgery was found as a risk factor for postoperative complications. Other parameters that showed significance in our scoring system were the stage, ECOG, ascites, major pelvic surgery, total surgical time, and diabetes. Preoperative albumin levels, CA-125 levels, COPD, and heart and renal disease were parameters that were assessed in prior studies^(4,6,7)^. These parameters have been found to be correlated significantly with postoperative complications. According to a study designed by Ataseven et al.^(23)^ preoperative hypoalbuminemia had been found as an independent predictive parameter for severe postoperative complications in epithelial ovarian cancer. Conversely, in our study, no significant correlation was found between hypoalbuminemia and postoperative complications in gynecologic cancers. CA-125 levels had no significant correlation with postoperative complications, which was probably influenced by our study design with the inclusion of all gynecologic cancers. Also, there were not many patients with COPD, renal disease, and heart disease in the study population, and thus these parameters were not found eligible for predicting complications. In our study, after estimating the risk assessment with ROC analysis, we found the AUC as 0.60 for the presence of complications and 0.70 for the number of complications, respectively. We have arranged a simple, practical and convenient model for preoperative risk assessment in patients with gynecological cancer.

### Study Limitations

The main strength of this study is the recommendation of a simple, practical, and convenient scoring model for preoperative risk assessment in patients with gynecologic cancer, also the addition of metoprolol use and upper abdominal surgery to preoperative risk assessment parameters.

Our study covered all gynecologic cancers and this is the main limitation of this study, Thus, to develop more effective scoring systems, further studies with specific patients and diagnostic groups are needed.

### Preclinical/clinical implications

In this research study we investigated if any preclinical/clinical implications would forebode postoperative complications. As a result, the evaluation and prediction of metoprolol use, upper abdominal surgery, stage of disease, ECOG, ascites, major pelvic surgery, total surgical time, and diabetes status were found as effective parameters; thus, preoperative improvement of these parameters could be beneficial in terms of reducing postoperative complications in gynecologic cancers.

## Conclusion

Several studies have shown that the use of perioperative metoprolol reduces mortality and morbidity in patients with both high and low cardiac risk. We added metoprolol use and upper abdominal surgery into the parameters of the evaluation system and as a result, metoprolol use decreased and upper abdominal surgery increased the risk and number of complications in gynecological cancers; therefore, these two parameters can also be used for predicting risk in patients with gynecologic cancer. Moreover, we have suggested a simple, practical and convenient scoring model for preoperative risk assessment in patients with gynecologic cancer.

## Figures and Tables

**Table 1:**
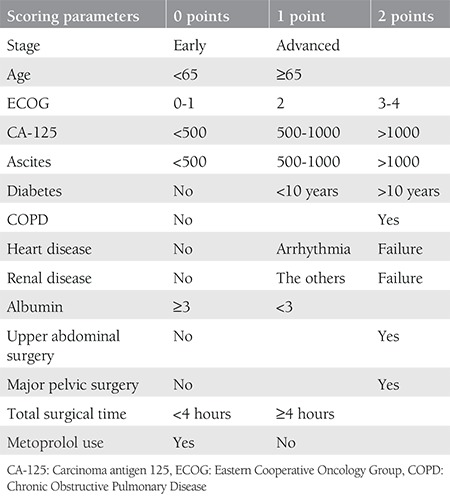
Scoring parameters and model

**Table 2:**
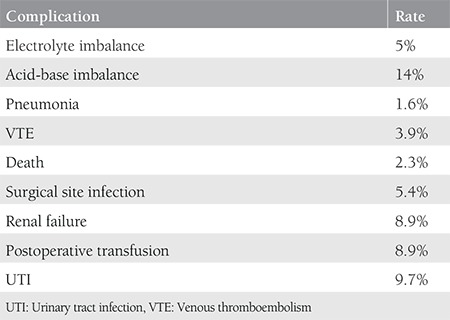
Complications and their distributions

**Table 3:**
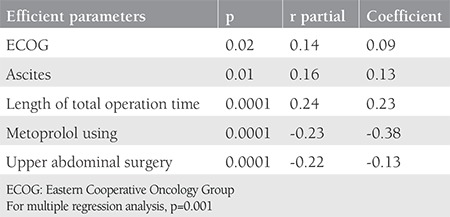
Multiple regression analysis results for the correlation between parameters and presence of complication (parameters with p<0.05 were included)

**Table 4:**
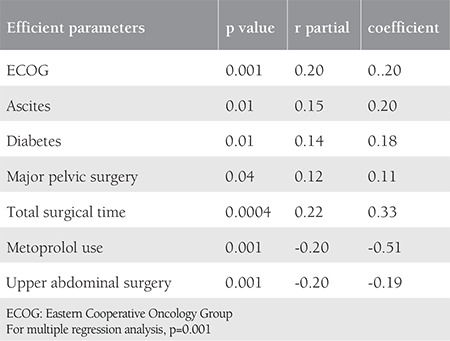
Multiple regression analysis results for the correlation between parameters and count of complications (parameters with p<0.05 were included)
